# Supplementary material for: Characterization and modulation of human insulin degrading enzyme conformational dynamics to control enzyme activity
Source: eLife. 2026 Jun 8;14:RP105761. doi: 10.7554/eLife.105761 (PMC13246006; doi:10.7554/eLife.105761)
Supplement: Supplementary file 1. [file elife-105761-supp1.docx]

| **Data collection and processing** | | | | | |
| --- | --- | --- | --- | --- | --- |
| Microscope | Titan Krios | | | | |
| Camera | Gatan K3 | | | | |
| Automation software | Leginon | | | | |
| Magnification | 81,000 | | | | |
| Voltage (kV) | 300 | | | | |
| Frames collected per micrograph | 50 | | | | |
| Dose per frame (e-/Å2) | 1.36 | | | | |
| Total electron dose (e-/Å2) | 67.9 | | | | |
| Defocus range (µM) | 0.7 to 1.5 | | | | |
| Total micrographs | 7,611 | | | | |
| Initial particle images (no.) | 7,206,464 | | | | |
|  | **O/O state** | **O/pO state** | **pO/pC state** | **O/pC state** | **pC/pC state** |
| Pixel size (Å) | 1.0842 | 1.0842 | 1.0842 | 1.0842 | 1.0842 |
| Final particle images (no.) | 77,973 | 328,870 | 76,379 | 304,011 | 1,341,061 |
| Symmetry imposed | C1 | C1 | C1 | C1 | C1 |
| Map resolution (Å) | 3.8 | 4.1 | 3.3 | 3.4 | 3.0 |
| FSC threshold | 0.143 | 0.143 | 0.143 | 0.143 | 0.143 |
| EMDB | EMD-24760 | EMD-24759 | EMD-24757 | EMD-24758 | EMD-24761 |
| **Refinement** | | | | | |
| Model resolution | | | | | |
| FSC 0.5 | 7.4 (7.7)^a^ | 4.5 (7.4)^a^ | 3.5 (3.8)^a^ | 3.6 (3.9)^a^ | 3.1 (3.2)^a^ |
| FSC 0.143 | 3.6 (3.8)^a^ | 3.4 (3.8)^a^ | 3.1 (3.2)^a^ | 3.3 (3.3)^a^ | 3.0 (3.0)^a^ |
| Sharpening B factor | -73.0 | -70.0 | -87.1 | -47.7 | -109.8 |
| Refinement package | PHENIX & COOT | PHENIX & COOT | PHENIX & COOT | PHENIX & COOT | PHENIX & COOT |
| Model composition | | | | | |
| Protein residues | 1867 | 1888 | 1898 | 1902 | 1926 |
| Total atoms | 15300 | 15465 | 15545 | 15584 | 15775 |
| B factors | | | | | |
| Protein | 64.38 | 129.58 | 70.19 | 80.87 | 26.93 |
| RMS deviations | | | | | |
| Bond length | 0.006 | 0.006 | 0.005 | 0.005 | 0.005 |
| Bond angle | 1.148 | 1.126 | 1.031 | 1.062 | 0.985 |
| Ramachandran (%) | | | | | |
| Favored | 96.99 | 94.36 | 96.34 | 95.92 | 97.07 |
| Allowed | 3.01 | 5.64 | 3.66 | 4.08 | 2.93 |
| Outliers | 0 | 0 | 0 | 0 | 0 |
| **Validation** | | | | | |
| MolProbity score | 1.51 | 1.67 | 1.34 | 1.49 | 1.3 |
| Poor rotamers (%) | 0.24 | 0.06 | 0.18 | 0.12 | 0.23 |
| Clash score | 6.07 | 5.26 | 3.03 | 4.25 | 3.44 |
| Correlation coefficient | 0.68 | 0.68 | 0.77 | 0.74 | 0.79 |
| Cbeta outliers | 0.11 | 0 | 0 | 0.06 | 0.05 |
| CaBLAM outliers | 1.89 | 2.73 | 1.76 | 1.86 | 1.9 |
| EMRinger score | 0.51 | 1.27 | 2.52 | 2.28 | 2.92 |
| PDB ID | 7RZH | 7RZG | 7RZE | 7RZF | 7RZI |
| ^a^Unmasked resolution is given in parentheses | | | | | |
