# Supplementary material for: Characterization and modulation of human insulin degrading enzyme conformational dynamics to control enzyme activity
Source: eLife. 2026 Jun 8;14:RP105761. doi: 10.7554/eLife.105761 (PMC13246006; doi:10.7554/eLife.105761)
Supplement: Supplementary file 2. [file elife-105761-supp2.docx]

| **Data collection and processing** | |
| --- | --- |
| Microscope | Titan Krios |
| Camera | Gatan K3 |
| Automation software | Leginon |
| Magnification | 81,000 |
| Voltage (kV) | 300 |
| Frames collected per micrograph | 50 |
| Dose per frame (e-/Å2) | 1.5 |
| Total electron dose (e-/Å2) | 65 |
| Defocus range (µM) | -0.7 to -2.5 |
| Total micrographs | 10,922 |
| Initial particle images (no.) | 12,985,439 |
|  | **O/O state** |
| Pixel size (Å) | 1.06 |
| Energy filter | 20 eV slit |
| Final particle images (no.) | 376,750 |
| Symmetry imposed | C1 |
| Map resolution (Å) | 5.15 |
| FSC threshold | 0.143 |
| EMDB | EMD-72393 |
| **Refinement** | |
| Model resolution | |
| FSC 0.5 | 7.2 (7.2)^a^ |
| FSC 0.143 | 4.9 (4.9)^a^ |
| Sharpening B factor | -270.4 |
| Refinement package | PHENIX & COOT |
| Model composition | |
| Protein residues | 1888 |
| Total atoms | 15412 |
| B factors | |
| Protein | 258.04 |
| RMS deviations | |
| Bond length | 0.003 |
| Bond angle | 0.755 |
| Ramachandran (%) | |
| Favored | 94.36 |
| Allowed | 5.54 |
| Outliers | 0.11 |
| **Validation** | |
| MolProbity score | 2.18 |
| Poor rotamers (%) | 0.06 |
| Clash score | 19.85 |
| Cbeta outliers | 0 |
| CaBLAM outliers | 2.84 |
| EMRinger score | 0.26 |
| PDB ID | 9Y0H |
| ^a^Unmasked resolution is given in parentheses | |
