# Supplementary material for: Characterization and modulation of human insulin degrading enzyme conformational dynamics to control enzyme activity
Source: eLife. 2026 Jun 8;14:RP105761. doi: 10.7554/eLife.105761 (PMC13246006; doi:10.7554/eLife.105761)
Supplement: Supplementary file 3. [file elife-105761-supp3.docx]

| **Component vector** | **Variance described (%)** | **Change in O state D1-D4 COM distance (Å)** | **Change in O state D1-D2-D3-D4 dihedral (degrees)** | **Change in pO state D1-D4 COM distance (Å)** | **Change in pO state D1-D2-D3-D4 dihedral (degrees)** |
| --- | --- | --- | --- | --- | --- |
| 1 | 12.59 | -4.1 | 20.2 | -1.3 | -19.2 |
| 2 | 11.22 | 4.1 | -2.9 | -10.4 | 14.2 |
| 3 | 10.23 | -21.2 | -8.8 | 5.5 | 3.1 |
| 4 | 7.77 | 17.4 | -33.9 | -1.6 | -1.2 |
| 5 | 7.39 | 12 | 2.8 | 5.3 | 6.6 |
| 6 | 6.97 | -5.8 | -9.9 | 2.6 | -7.7 |
| 7 | 6.71 | -16.3 | -26 | -0.4 | 5.7 |
| 8 | 6.2 | -3.3 | -0.7 | 2.3 | -11.5 |
| 9 | 5.73 | 2.1 | -3.4 | -0.3 | 1.8 |
