# Supplementary material for: Characterization and modulation of human insulin degrading enzyme conformational dynamics to control enzyme activity
Source: eLife. 2026 Jun 8;14:RP105761. doi: 10.7554/eLife.105761 (PMC13246006; doi:10.7554/eLife.105761)
Supplement: Supplementary file 4. [file elife-105761-supp4.docx]

| **Component vector** | **Variance described (%)** | **Change in O state D1-D4 COM distance (Å)** | **Change in O state D1-D2-D3-D4 dihedral (degrees)** | **Change in pO state D1-D4 COM distance (Å)** | **Change in pO state D1-D2-D3-D4 dihedral (degrees)** |
| --- | --- | --- | --- | --- | --- |
| 1 | 16.4 | -1.5 | 5.8 | 3.7 | -7.6 |
| 2 | 15.5 | -1.3 | -22.6 | 1.1 | -4 |
| 3 | 12.1 | 14.3 | -6.1 | 0.2 | 4.4 |
| 4 | 11.9 | -7 | 3.5 | 0.3 | 10.9 |
| 5 | 7.37 | -1.5 | -4.8 | -0.1 | 0.8 |
| 6 | 6.25 | 1 | 14 | -0.7 | -11 |
| 7 | 5.85 | 0.1 | 0.6 | 3 | -2 |
| 8 | 4.95 | -1.4 | 1.2 | 1 | -2.1 |
| 9 | 4.19 | 2.8 | 5.8 | -0.5 | -6 |
