# Supplementary material for: Characterization and modulation of human insulin degrading enzyme conformational dynamics to control enzyme activity
Source: eLife. 2026 Jun 8;14:RP105761. doi: 10.7554/eLife.105761 (PMC13246006; doi:10.7554/eLife.105761)
Supplement: Supplementary file 5. [file elife-105761-supp5.docx]

| **Component vector** | **Variance described (%)** | **Change in O_1_ state D1-D4 COM distance (Å)** | **Change in O_1_ state D1-D2-D3-D4 dihedral (degrees)** | **Change in O_2_ state D1-D4 COM distance (Å)** | **Change in O_2_ state D1-D2-D3-D4 dihedral (degrees)** |
| --- | --- | --- | --- | --- | --- |
| 1 | 17.6 | 5.3 | -7.4 | 0.4 | 1.4 |
| 2 | 15.9 | -18 | -27.8 | -0.8 | -5.3 |
| 3 | 13 | 0 | 4.6 | -12.2 | -16.5 |
| 4 | 11.7 | 0.1 | 2.4 | 15 | -21.1 |
| 5 | 8.52 | -0.7 | -11.2 | -2.5 | 11.4 |
| 6 | 8.21 | -0.3 | -10.8 | 5.7 | 9 |
| 7 | 7.12 | 5.2 | -0.7 | -1.9 | 1 |
| 8 | 5.45 | -18 | -16.3 | -1.2 | 13.1 |
| 9 | 3.2 | -2.5 | 3 | 7.3 | -4.3 |
