# Supplementary material for: Characterization and modulation of human insulin degrading enzyme conformational dynamics to control enzyme activity
Source: eLife. 2026 Jun 8;14:RP105761. doi: 10.7554/eLife.105761 (PMC13246006; doi:10.7554/eLife.105761)
Supplement: Supplementary file 6. [file elife-105761-supp6.docx]

| **Component vector** | **Variance described (%)** | **Change in O state D1-D4 COM distance (Å)** | **Change in O state D1-D2-D3-D4 dihedral (degrees)** | **Change in pC state D1-D4 COM distance (Å)** | **Change in pC state D1-D2-D3-D4 dihedral (degrees)** |
| --- | --- | --- | --- | --- | --- |
| 1 | 15.8 | 6.8 | -15.6 | -3.4 | 1.2 |
| 2 | 13.8 | 0.1 | -4.8 | 5.4 | -20.3 |
| 3 | 12.1 | -0.1 | -1.9 | 11.4 | 0.9 |
| 4 | 11.2 | 1.8 | 22.6 | -0.2 | -5.1 |
| 5 | 10.1 | -0.6 | -8.8 | 1.7 | 12.2 |
| 6 | 7.91 | 0.1 | 1.9 | 0.6 | -10.9 |
| 7 | 7.04 | 2.3 | -9.9 | -0.8 | 0.8 |
| 8 | 6.29 | 5.6 | -2.5 | -2.2 | 3.4 |
| 9 | 5.07 | -0.8 | 3.9 | 4.9 | -6.3 |
