# Supplementary material for: Characterization and modulation of human insulin degrading enzyme conformational dynamics to control enzyme activity
Source: eLife. 2026 Jun 8;14:RP105761. doi: 10.7554/eLife.105761 (PMC13246006; doi:10.7554/eLife.105761)
Supplement: Supplementary file 7. [file elife-105761-supp7.docx]

| **Component vector** | **Variance described (%)** | **Change in pC_1_ state D1-D4 COM distance (Å)** | **Change in pC_1_ state D1-D2-D3-D4 dihedral (degrees)** | **Change in pC_2_ state D1-D4 COM distance (Å)** | **Change in pC_2_ state D1-D2-D3-D4 dihedral (degrees)** |
| --- | --- | --- | --- | --- | --- |
| 1 | 17.1 | 7 | -16 | -1.7 | -0.8 |
| 2 | 13.9 | 1.6 | 19.3 | 0.7 | 3.3 |
| 3 | 13.6 | 0.1 | 5.3 | -6.7 | -8.2 |
| 4 | 10.8 | -0.2 | 1 | 9.8 | -13.4 |
| 5 | 8.16 | -2.7 | -10.7 | 2.8 | 13.1 |
| 6 | 8.06 | 4.1 | -7.5 | 0.3 | 1 |
| 7 | 6.49 | 2.1 | -2.8 | -2.5 | 11.6 |
| 8 | 4.81 | 1.1 | -10.1 | -1 | 1.4 |
| 9 | 3.69 | -2.5 | 3.4 | 5.6 | -6.6 |
