# Supplementary material for: Characterization and modulation of human insulin degrading enzyme conformational dynamics to control enzyme activity
Source: eLife. 2026 Jun 8;14:RP105761. doi: 10.7554/eLife.105761 (PMC13246006; doi:10.7554/eLife.105761)
Supplement: Supplementary file 8. [file elife-105761-supp8.docx]

| **Component vector** | **Variance described (%)** | **Change in pO state D1-D4 COM distance (Å)** | **Change in pO state D1-D2-D3-D4 dihedral (degrees)** | **Change in pC state D1-D4 COM distance (Å)** | **Change in pC state D1-D2-D3-D4 dihedral (degrees)** |
| --- | --- | --- | --- | --- | --- |
| 1 | 24.9 | 4.6 | -9.1 | 0.6 | -0.3 |
| 2 | 17 | -0.1 | 1.9 | 8.2 | -11.4 |
| 3 | 12.5 | -1 | -10.9 | 1.6 | -1.5 |
| 4 | 8.92 | 0.7 | -0.2 | 2.1 | 6.2 |
| 5 | 8.54 | -1.2 | 3.5 | 1.7 | 3 |
| 6 | 6.15 | -0.1 | -1.4 | 0 | -5 |
| 7 | 3.98 | 0.4 | 7.4 | -1 | -7.6 |
| 8 | 2.79 | 1.1 | -2.9 | -1 | -0.6 |
| 9 | 2.31 | -2.3 | 2 | 3.1 | -3.7 |
