# Supplementary material for: Characterization and modulation of human insulin degrading enzyme conformational dynamics to control enzyme activity
Source: eLife. 2026 Jun 8;14:RP105761. doi: 10.7554/eLife.105761 (PMC13246006; doi:10.7554/eLife.105761)
Supplement: Peer review history

# Editor report: eLife Assessment

**DOI:** 10.7554/eLife.105761.4.sa0

**Authors:** Julien Roche

**Affiliations:** Iowa State University United States

**Keywords:** Compelling, Important

The manuscript by Mancl et al. provides **important** mechanistic insights into the conformational dynamics of Insulin Degrading Enzyme (IDE), a zinc metalloprotease involved in the clearance of amyloid peptides. Supported by a **compelling** combination of time-resolved cryo-EM, SEC-SAXS, enzymatic assays, and both all-atom and coarse-grained simulations, the study reveals an insulin-induced allosteric transition and transient β-sheet interactions underlying IDE's unfoldase activity, thereby refining our understanding of IDE's functional cycle and offering a structural framework for developing substrate-selective modulators of M16 metalloproteases. The latest round of revisions further improves clarity and presentation by updating structural statistics, correcting minor textual inconsistencies, and refining supplemental materials, fully addressing the remaining reviewer comments.

# Referee report: Reviewer #1 (Public review)

**DOI:** 10.7554/eLife.105761.4.sa1

Summary:

Mancl et al. present an integrative structural and mechanistic analysis of the human insulin-degrading enzyme (IDE), combining cryo‑EM, time‑resolved cryo‑EM, SEC‑SAXS, enzymatic assays, all-atom molecular dynamics (MD) simulations, and coarse‑grained MD simulations. Their study delineates how IDE undergoes coordinated open-close transitions and interdomain rotations, how these motions relate to its unfoldase and protease activities, and how a single residue, R668, acts as a molecular latch governing these conformational changes. Through expanded structural datasets and computational analyses, the authors propose a mechanistic model for how IDE captures, unfolds, and degrades diverse amyloidogenic substrates such as insulin and Aβ.

Strengths:

A major strength of this study is its integration of structural, biophysical, biochemical, and computational approaches. The authors now provide six cryo‑EM structures, including a new time‑resolved O/O state captured 123 ms after substrate mixing, which clarifies the early structural response of IDE to insulin binding. The combination of multibody analysis, 3D variability analysis, all‑atom MD, and coarse‑grained Upside simulations yields a coherent picture in which rotational interdomain motions and charge‑swapping events at the IDE‑N/C interface underpin substrate unfolding and repositioning.

The identification of R668 as a central determinant of the open-close transition, supported by MD, HDX‑MS data from prior work, SEC‑SAXS, and functional assays on the R668A mutant, represents a significant mechanistic advance. The inclusion of Aβ degradation assays adds biological breadth and supports the conclusion that R668 modulates activity in a substrate‑dependent manner.

The authors have also substantially improved clarity by reorganizing figures, refining section headers, and adding introductory structural schematics. Taken together, the revised manuscript now provides a rigorous and accessible framework for understanding IDE dynamics and their relevance to amyloid peptide turnover.

Weaknesses:

At this stage, remaining limitations are modest and inherent to the system rather than the approach. While the study convincingly demonstrates substrate‑dependent modulation of IDE dynamics, it does not experimentally assess additional endogenous substrates (e.g., amylin, glucagon), which would be needed to fully generalize the role of R668 across the substrate spectrum of IDE. Furthermore, the timescale mismatch between MD simulations and catalytic turnover, which the authors clearly acknowledge, means that correlations between simulated motions and enzymatic kinetics remain inferential. Finally, some flexible cryo‑EM states (particularly O/pO) continue to exhibit moderate local resolution, which constrains atomic interpretation of highly dynamic regions, although this is addressed transparently.

# Referee report: Reviewer #2 (Public review)

**DOI:** 10.7554/eLife.105761.4.sa2

Summary:

The manuscript describes various conformational states and structural dynamics of the Insulin degrading enzyme (IDE), a zinc metalloprotease by nature. Both open and closed state structures of IDE have been previously solved using crystallography and cryo-EM which reveal a dimeric organization of IDE where each monomer is organized into N and C domains. C-domains form the interacting interface in the dimeric protein while the two N-domains are positioned on the outer sides of the core formed by C-domains. It remains elusive how the open state is converted into the closed state but it is generally accepted that it involves large-scale movement of N-domains relative to the C-domains. Authors here have used various complementary experimental techniques such as cryo-EM, SAXS, size-exclusion chromatography and enzymatic assays to characterize the structure and dynamics of IDE protein in the presence of substrate protein insulin whose density is captured in all the structures solved. The experimental structural data from cryo-EM suffered from high degree of intrinsic motion amongst the different domains and consequently, the resultant structures were moderately resolved at 3-4.1 Å resolution. Total five structures were generated in the originally submitted manuscript using cryo-EM. Another cryo-EM reconstruction (sixth) at 5.1Å resolution was mentioned after first revision which was obtained using time-resolved cryo-EM experiments. Authors have extensively used Molecular dynamics simulation to fish out important inter-subunit contacts which involves R668, E381, D309, etc residues. In summary, authors have explored the conformational dynamics of IDE protein using experimental approaches which are complemented and analyzed in atomic detail by using MD simulation studies. The studies are meticulously conducted and lay the ground for future exploration of the protease structure-function relationship.

Strengths:

The manuscript presents a powerful integrative structural biology study that combines high-resolution cryo-EM, particle heterogeneity analysis, time-resolved cryo-EM, multiscale molecular dynamics simulations, SAXS, and biochemical assays to dissect the conformational dynamics of human insulin-degrading enzyme. A major strength is the identification of a previously unappreciated rotational component of IDE-N relative to IDE-C and the discovery of R668 as a molecular latch governing the open-close transition, supported consistently by structural, computational, mutational, and functional data. The work provides a coherent mechanistic framework linking IDE dynamics to substrate unfolding, allostery, and substrate-dependent catalysis, with clear relevance to diabetes and Alzheimer's disease biology.

Weaknesses:

Despite its depth, several key mechanistic conclusions-particularly substrate unfolding and the proposed "β-grabbing" mechanism-rely heavily on coarse-grained and all-atom MD simulations rather than direct experimental observation. Cryo-EM density for insulin is limited and heterogeneous, restricting definitive structural interpretation of substrate binding modes. The time-resolved cryo-EM experiment captures only a single dominant state at modest resolution, limiting insight into transient intermediates. In addition, the study focuses primarily on insulin, leaving the generality of the proposed mechanism for other IDE substrates insufficiently tested, and the therapeutic implications remain largely speculative without direct pharmacological modulation data.

# Author comment: Author response

**DOI:** 10.7554/eLife.105761.4.sa3

**Authors:** Jordan M Mancl, Wenguang G Liang, Nicholas L Bayhi, Hui Wei, William C Budell, Jashua H Mendez, Tobin R Sosnick, Bridget Carragher, Clinton S Potter, Wei-Jen Tang

**Affiliations:** University of Chicago Chicago United States; University of Chicago Chicago United States; University of Chicago Chicago United States; New York Structural Biology Center New York United States; New York Structural Biology Center New York United States; New York Structural Biology Center New York United States; University of Chicago Chicago United States; Chan Zuckerberg Imaging Institute Redwood, CA United States; Chan Zuckerberg Imaging Institute Redwood, CA United States; University of Chicago Chicago United States

The following is the authors’ response to the previous reviews

**Public Reviews:**

**Reviewer #1 (Public review):**

Summary:

Mancl et al. present a comprehensive integrative study combining cryo-EM, SAXS, enzymatic assays, and molecular dynamics (MD) simulations to characterize conformational dynamics of human insulin-degrading enzyme (IDE). In the revised manuscript, the study now also includes time-resolved cryo-EM and coarse-grained MD simulations, which strengthen the mechanistic model by revealing insulin-induced allostery and β-sheet interactions between IDE and insulin. Together, these results expand the original mechanistic insight and further validate R668 as a key residue governing the open-close transition and substrate-dependent activity modulation of IDE.

Strengths:

The authors have substantially expanded the experimental scope by adding time-resolved cryo-EM data and coarse-grained MD simulations, directly addressing requests for mechanistic depth and temporal insight. The integration of multiple resolution scales (cryo-EM heterogeneity analysis, all-atom and coarse-grained MD simulations, and biochemical validation) now provides a coherent description of the conformational transitions and allosteric regulation of IDE. The addition of Aβ degradation assays strengthens the claim that R668 modulates IDE function in a substrate-specific manner. Finally, the manuscript reads more clearly: figure organization, section headers, and inclusion of a new introductory figure make it accessible to a broader audience. Overall, the revision reinforces the conceptual advance that the dynamic interdomain motions of IDE underlie both its unfoldase and protease activities and identifies structural motifs that could be targeted pharmacologically.

Weaknesses:

While the authors acknowledge that future studies on additional IDE substrates (e.g., amylin and glucagon) are warranted, such experiments remain outside the present scope. Their absence modestly limits the generalization of the R668 mechanism across all IDE substrates. Despite improved discussion of kinetic timescales and enzyme-substrate interactions, experimental correlation between MD timescales and catalysis remains primarily inferential. The moderate local resolution of some cryo-EM states (notably O/pO) continues to limit atomic interpretation of the most flexible regions, though the authors address this carefully.

**Reviewer #2 (Public review):**

Summary:

The manuscript describes various conformational states and structural dynamics of the Insulin degrading enzyme (IDE), a zinc metalloprotease by nature. Both open and closed state structures of IDE have been previously solved using crystallography and cryo-EM which reveal a dimeric organization of IDE where each monomer is organized into N and C domains. C-domains form the interacting interface in the dimeric protein while the two N-domains are positioned on the outer sides of the core formed by C-domains. It remains elusive how the open state is converted into the closed state but it is generally accepted that it involves large-scale movement of N-domains relative to the C-domains. Authors here have used various complementary experimental techniques such as cryo-EM, SAXS, size-exclusion chromatography and enzymatic assays to characterize the structure and dynamics of IDE protein in the presence of substrate protein insulin whose density is captured in all the structures solved. The experimental structural data from cryo-EM suffered from high degree of intrinsic motion amongst the different domains and consequently, the resultant structures were moderately resolved at 3-4.1 Å resolution. Total five structures were generated in the originally submitted manuscript using cryo-EM. Another cryo-EM reconstruction (sixth) at 5.1Å resolution was mentioned after first revision which was obtained using time-resolved cryo-EM experiments. Authors have extensively used Molecular dynamics simulation to fish out important inter-subunit contacts which involves R668, E381, D309, etc residues. In summary, authors have explored the conformational dynamics of IDE protein using experimental approaches which are complimented and analyzed in atomic details by using MD simulation studies. The studies are meticulously conducted and lay ground for future exploration of protease structure-function relationship.

Comments after first peer-review:

The authors have addressed all my concerns, and have added new data and explanations in terms of time-resolved cryo-EM (Fig. 7) and upside simulations (Fig. 8) which in my opinion have strengthened the merit of the manuscript.

We are grateful for the dedication and constructive feedback provided by the editors and reviewers. We have revised our manuscript according to the suggestions by both reviewers.

**Recommendations for the authors:**

**Reviewer #1 (Recommendations for the authors):**

The new version of the manuscript reads exceedingly well and the corrections the authors have made during their revision made the manuscript much easier to read and digest than the first version. Below are minor details that may be corrected:

Abstract:

Line 45-47: "IDE is known to transition between a closed state, poised for catalysis, and an open state, able to release cleavage products and bind a new substrate." (consider adding a)

Fixed

Line 48-50: "Combining cryo-EM heterogeneity analysis with all-atom molecular dynamics (MD) simulations, we identified the structural basis and key residues for IDE conformational dynamics that were not previously revealed by IDE static structures." (consider adding previously)

Changed

Line 52-54: "Our small-angle X-ray scattering analysis and enzymatic assays of an R668A mutant indicate a profound alteration of conformational dynamics and catalytic activity." (consider adding analysis)

Changed

Line 54: Consider leaving out "Upside" in the abstract (to avoid confusion when reading the abstract) and leave it to be introduced in the introduction when Upside MD simulations are first mentioned.

Changed

Results:

Figure 5D: There seems to be an error in the legend for Figure 5D. It says "... presence of varying amounts of insulin", but this must be Aβ1-40. Please add info on whether the replicates are technical or biological.

The legend has been revised as suggested.

Line 125: Consider switching the order of "here" and "we"

“here” has been removed.

Line 128: Replace "5" with "five"

Changed

Line 137: Replace "when insulin is present" with "in the presence of insulin"

Changed

Line 228: Replace "5" and "6" with "five " and "six"

Changed

Line 229: Consider adding the word "form": "First, the open subunits did not close to form a singular structure."

We have adjusted the sentence to read “close to a singular consensus structure”

Line 327: Replace "2" with "two"

Changed

Line 276: Consider replacing "Conversely" with a more suitable connecting term as it implies that the observation presented in the two sentences are reverse or rephrase what is being compared. Is it the fact there is a dose dependency or not between the substrates or is it the actual kinetic parameters that are described. I just don't think conversely is fair with the current formulation as "the R668A mutant did not exhibit a dose-dependent response to the presence of Aβ" not that the Ki is reduced for WT compared to the R668A construct when looking at Aβ.

The connecting term has been removed completely, beginning the sentence with “When Abeta…”

Line 359: Replace "6" with "six"

Changed

Consider getting rid of possessive apostrophes to keep a formal tone, e.g. lines 211 (cryoSPARC's), 259 (IDE's) and 382 (IDE's). Exception to this is Alzheimer's disease.

All instances of possessive apostrophes, aside from Alzheimer’s, have been replaced alter more formal wording.

Figure 7 supplement 1: The color scheme for the local resolution is missing the unit (Å).

This has been corrected.

Finally, the supplementary videos illustrating IDE conformational dynamics are difficult to interpret and somewhat redundant in their current form. The transitions occur very rapidly, making it hard to appreciate the described motions, and the uniform coloring of IDE further limits visual clarity. I apologize for not including this point in my initial review. I recommend either removing the videos or re-rendering them to improve interpretability, for example by slowing down the motion and applying the same domain color scheme introduced in the new Figure 1 (and used in the MD trajectory video). This would greatly aid readers in connecting the descriptions in the text to the visual representations in the movies.

Figure 3 videos 1-4 were slowed down, simplified, and recolored to improve clarity.

**Reviewer #2 (Recommendations for the authors):**

Comments after first revision for authors:

Thanks a ton to the authors for the detailed explanation on my comments. I believe the discussions will help a large group of audience, especially the non-experts. Please address the minor comment below:

Minor comment:

Please update Supplementary file 1 (Cryo-EM data collection, refinement, and validation statistics) regarding the new volume obtained by time-resolved cryo-EM. Kindly also check line 47 in the abstract: "Here, we present five cryo-EM structures" , which may need an update (six structures and resolution 3.0-5.1 Å) or rephrase the sentences accordingly. If similar instances are found in the manuscript, where list of all the structures are mentioned together, please update accordingly if necessary.

The cryo-EM statistics for the time-resolved cryo-EM are shown in supplementary file 2 to differentiated two datasets. The abstract has been changed, as has line 149.
